# Iron removal from red clay using oxalic acid leaching for enhanced ceramic industry applications

**DOI:** 10.1016/j.heliyon.2024.e38863

**Published:** 2024-10-04

**Authors:** Hayatullah, Asma Shafiq Shathi, Md Golam Mostafa, Md Aminur Rahman, Pradip Kumar Biswas, Md Sha Alam, Md Shohel Rana, Md Ripaj Uddin, Md Nuruzzaman, Md Shams Shahriar, Mohammad Nazim Zaman

**Affiliations:** a Institute of Mining, Mineralogy and Metallurgy (IMMM), Bangladesh Council of Scientific and Industrial Research (BCSIR), Khanjonpur, Joypurhat, 5900, Bangladesh; b BCSIR Dhaka Laboratories, Bangladesh Council of Scientific and Industrial Research (BCSIR), Dhaka, Bangladesh

**Keywords:** Red clay, Bulk mineralogy, Iron oxides, Oxalic acid leaching and ceramics trial

## Abstract

This work focused on removing iron with oxalic acid from red clay samples collected from Kapasia Upazila, Gazipur District, Bangladesh. To characterize the red clay, the study employed several techniques: particle size analysis, WDXRF (wavelength-dispersive X-ray fluorescence), AAS (atomic absorption spectroscopy), XRD (X-ray diffraction), TGA (thermogravimetric analysis), and FESEM (field emission scanning electron microscopy). Additionally, leaching experiments were conducted with varying concentrations of oxalic acid, temperatures and times. After leaching, the red clay composition changed significantly: SiO₂ increased from 53.6 % to 63.13 %, Fe₂O₃ decreased from 17.1 % to 3.64 %, Al₂O₃ remained relatively stable at 18 %–18.22 %, and other oxides showed minor variations. 78.71 % of the iron was removed at optimal leaching conditions (1.0 M oxalic acid, 100 °C, 150 min, and 250 rpm). Mineralogically, the red clay samples are composed of illite, kaolinite, quartz, feldspar, hematite and chlorite. Thermal analysis showed significant weight loss at temperatures between 300 and 600 °C. Ceramic trials were conducted at firing temperatures of 900 °C and 1100 °C to evaluate the mechanical properties of tiles. The results obtained showed significant improvements in red clay quality for ceramics. Being a low-cost and eco-friendly process, this becomes a very prominent alternative to conventional iron removal techniques and helps produce high-quality ceramic tiles, contributing towards economic growth in Bangladesh.

## 1 Introduction

Kapasia Upazila in Gazipur District, Bangladesh, is a significant source of raw materials, particularly high-quality ceramic production. The red clay deposits through the weathering of feldspathic rocks and the subsequent accumulation of clay minerals [[1], [2], [3]].

Red clay is widely utilized as a raw material in pottery, ceramics, paper, rubber, and various other industries [4]. The presence of impurities such as silica, mica, and iron oxides significantly impacts the quality of the final ceramic products [[5], [6], [7]]. Among these impurities, iron is abundant in an insoluble state in nature, which poses a particular challenge as high iron content prevents the production of high-quality ceramic tiles [8]. To achieve good quality ceramic tiles, the iron content in red clay samples should be less than 0.8 % by weight [9,10]. Froth flotation, acid treatment, reductive roasting, gravity and magnetic separation are traditional methods used to determine the beneficiation of red clay [11,12]. While many ceramic industries have adopted and refined these methods for ceramic trials, they are highly energy-intensive, costly, and contribute to environmental pollution [13]. Flotation techniques are high maintenance and reagent-intensive and frequently produce large amounts of froth waste. Analogously, significant emissions of gasses and particles are released during reductive roasting, which further contributes to environmental contamination [14]. Conversely, hydrofluoric, hydrochloric, sulfuric, and perchloric acid leaching methods are more environmentally safe, cost-effective, less complex, but remove less iron from red clay [15]. In addition, organic acids such as oxalic, citric, ascorbic, gluconic, and malic acids are more effective than inorganic acids at removing iron impurities from red clay [16]. Among these, oxalic acid stands out due to its strong acidity, high reducing power, and efficiency in iron removal [17]. Liu et al. [8] investigated the leaching efficiencies of aluminum, silicon, and titanium using 1.0 mol/L oxalic acid at 80 °C, after 2 h, the leaching efficiencies ranged from 63 % to 75 %. Additionally, several studies on the removal of iron from red clay have been conducted, as summarized in Table 1.

**Table 1:** Background study of leaching parameters.

The initial amount of Fe_2_O_3_	Leaching chemicals	Suitable Parameter			Fe_2_O_3_ (wt%)	References
		Leaching time (min.)	Acid conc. (M)	Temperature (^o^C)		
Fe_2_O_3,_ 0.93 %	Oxalic, ascorbic, citric, formic, acetic and succinic acid.	90	0.15	100	73	[18]
Fe_2_O_3_, 0.18 %	Oxalic acid	120	0.5	100	20	[19]
Fe_2_O_3_, 0.07 %	Acetic, oxalic, citric, gluconic acid	120	1	90	34.5	[20]
Fe_2_O_3,_ 40 %	H_2_SO4_2_	120	3	90	37.0	[21]
Fe_2_O_3_, 3.09 %	Oxalic acid	120	0.5	85	79.9	[22]
Fe_2_O_3_,17.1 %	Oxalic acid	150	1	100	78.71	In this study

However, this research prioritizes oxalic acid due to its higher iron removal yield and eco-friendly nature compared to other acids [[23], [24], [25]]. The ceramics industry in Bangladesh is among the rapidly growing manufacturing sectors. After fulfilling 80 % of domestic demand, Bangladesh exported approximately US$36 million worth of ceramic goods. However, ceramic manufacturers largely depend on imported raw materials, primarily sourced from China, India, New Zealand, and Germany [26]. Local production meets about 95 % of the demand for tableware, 75 % for tiles, and 85 % for sanitary ware.

Despite extensive research on the beneficiation of red clay, there is a notable gap in the literature regarding the use of iron-free red clay for ceramic applications in Bangladesh. Previous studies have focused on the beneficiation processes and the chemical and physical properties of red clay but have not adequately explored the application of treated red clay in ceramic industries within the Bangladeshi context [27,28]. This study is justified by the need to enhance the quality of red clay for ceramic applications, thereby improving the economic viability and environmental sustainability of ceramic production in Bangladesh. By employing oxalic acid leaching, a method proven to be both effective and eco-friendly, this research aims to fill the gap in the existing literature and provide a practical solution for the ceramic industry. This approach not only addresses the iron impurity issue but also promotes the utilization of local clay resources, contributing to the development of the ceramic industry in Bangladesh.

## 2 Materials and methods

### 2.1 Sample collection and preparation

**Fig. 1:**
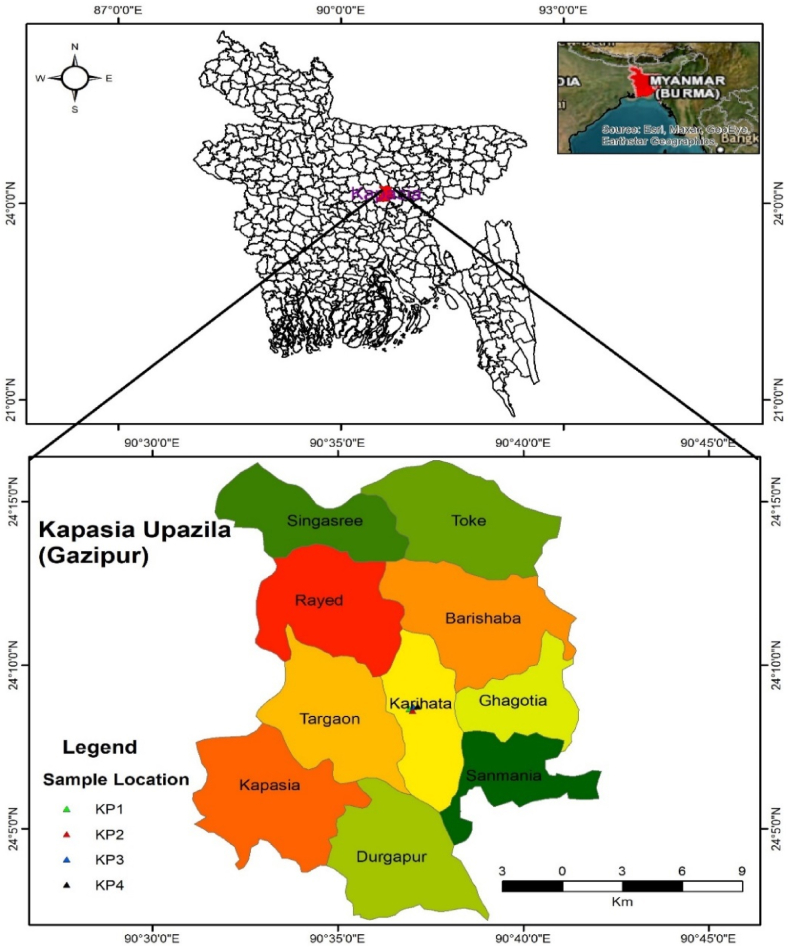
Location map of the Kapasia Upazila, Gazipur District, Bangladesh.

Fieldwork was conducted during the dry season (January 2022) and samples were collected from Kapasia Upazila, Gazipur District, Bangladesh. Geologically, the study area is bounded by longitudes 90°30ʹ0ʹʹ to 90°45ʹ0ʹʹ and latitudes 24°50ʹ0ʹʹ to 24°15ʹ0ʹʹ (Fig. 1). Approximately 3 kg of red clay were collected from four boreholes with depths of 100 feet, 105 feet, 110 feet, and 115 feet, respectively. The samples were then mixed in equal proportions to ensure consistency and uniform color.

### 2.2 Analytical analysis

For preparing the ceramic trials and characterization, the samples were air-dried for 24 h, ground with a hand mortar, and then sieved using a 250 μm mesh according to American Society for Testing and Materials (ASTM) standard methods. A laser diffraction particle analyzer (Microtrac Turbotrac, S = 3500) was employed to determine the particle size distribution of sand, silt, and clay. X-ray fluorescence (XRF) spectrometry (Rigaku ZSX Primus, 4 kW Rh-anode X-ray tube) was used to assess the chemical composition of the red clay samples. Atomic absorption spectrometry (Agilent 240FS AAS) was utilized to measure the concentration of leached red clay. X-ray diffraction (XRD) analysis (Panalytical XPERTPRO, PW3040/60, scanning rate of 2.4° 2θ/min) was performed to identify the crystalline phases within a scan range of 5–65° 2θ. Additionally, thermogravimetric analysis (PerkinElmer STA 8000, USA, with a nitrogen flow rate of 20 ml/min) was used to examine the thermal stability and weight loss percentage of the samples within a temperature range of 50–900 °C at a heating rate of 10 °C/min. The microstructure and elemental composition of the red clay samples were analyzed using field emission scanning electron microscopy with energy dispersive x-ray spectroscopy (FESEM-EDS, Sigma 300, Zeiss, Germany, equipped with a Bruker X Flash 6160 EDS detector).

### 2.3 Iron removal procedure

**Fig. 2:**
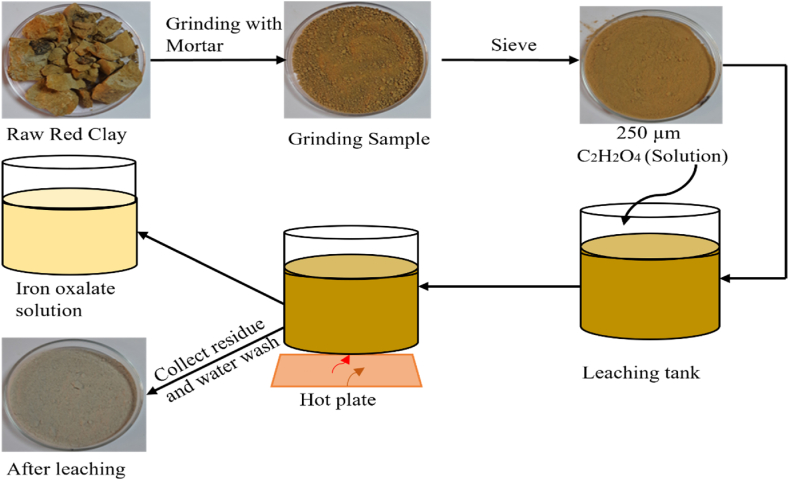
Schematic diagram of oxalic acid leaching of red clay.

The chemical leaching experiments were conducted in a 250 ml beaker that was agitated on a hot plate (Fig. 2). For each experiment, 100 ml of reagent-grade oxalic acid solution (C_2_H_2_O_4_) at varying concentrations was added to the beaker, and the temperature was adjusted to the desired level. Subsequently, 10 g of red clay was added to the beaker and stirred magnetically at 250 rpm. From the leach slurry, a 10 ml sample was taken and centrifuged for 10 min at 1000 rpm. To determine the total iron content removed from the solution, a clear 5 ml sample was collected. The final residue was washed with distilled water to remove any remaining acid matrix. The rinsed residues were then cooled after being oven-dried. Various concentrations of oxalic acid (0.10, 0.30, 0.50, 0.80, 1.0 M, and 2 M), reaction temperatures (40, 60, 80, and 100 °C), and leaching times (30, 60, 90, 120, and 150 min) were investigated to determine the optimal conditions for the removal of iron from the red clay samples.

The following stoichiometry reaction process is given below:

(1) H_2_C_2_O_4_ (aq) → H^+^ + HC_2_O_4_^−^

(2) HC_2_O_4_^−^ → H^+^ + C_2_O_4_^−^

(3) Fe_2_O_3_ + H^+^ + 5HC_2_O_4_^−^ → 2Fe (C_2_O_4_)_2_^2−^ + 3H_2_O + 2CO_2_

In the oxalic acid solution, eq. (1), the bi-oxalate ion (C_2_H_2_O_4_) is released when C_2_H_2_O_4_ (aq) dissociates. In equation (2), the bi-oxalate ion forms dissociate to produce oxalate ions (C_2_O_4_)^−2^. As demonstrated by eq. (3), it is clear from equations (1), (2) that the bioxalate is in charge of removing the iron impurities from the red clay surface.

### 2.4 Preparation of ceramic tiles

**Fig. 3:**
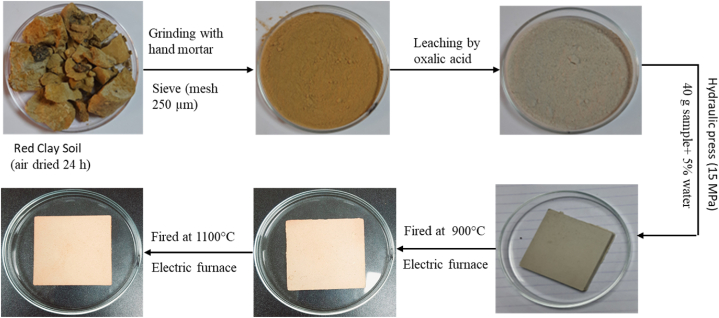
Flow chart for making the red clay ceramic tiles.

The procedure for making tiles from red clay involves several steps to ensure the quality and durability of the final product. First, 10 kg of red clay samples were selected based on their consistency and color. XRF, XRD, and other results revealed that the red clay samples are capable of being used in ceramics trials. The samples were air-dried for 24 h, ground using a hand mortar, and then sieved with a 250 μm mesh according to ASTM standard methods. Next, the sieved clay was mixed with water to achieve a workable consistency. After that, 40g samples were mixed with 5%–7% water to make rectangular test specimens (50 mm × 50 mm x 10 mm) and compressed at 15 MPa using a hydraulic hot press (Model 2–118, Tekcast, US). In this manner, approximately 250 tiles were prepared. The molded tiles were dried in an electric oven at 110 °C for 24 h (Electric oven, 110 °C to remove excess moisture. Following the drying process, the tiles were fired in an electric furnace (Protherm Furnace-1300 °C, PLF 130/45, Turkey) at various temperatures, typically 900 °C and 1100 °C, with a heating rate of 10 °C/min and a dwell time of 1 h (Fig. 3). This firing process enhances the strength and durability of the tiles by inducing vitrification and other chemical changes in the clay material. Finally, Archimedes' immersion technique was used to assess the firing parameters of the final ceramic tiles. Furthermore, unconfined compressive stress was examined using the unconfined compression apparatus (Proving Ring 5 kN). These tests ensure that the tiles meet industry standards for quality and performance. Through this detailed procedure, red clay can be effectively utilized to produce high-quality ceramic tiles suitable for various applications.

## 3 Results and discussion

### 3.1 Effect of oxalic acid concentration and temperature on leaching

**Fig. 4:**
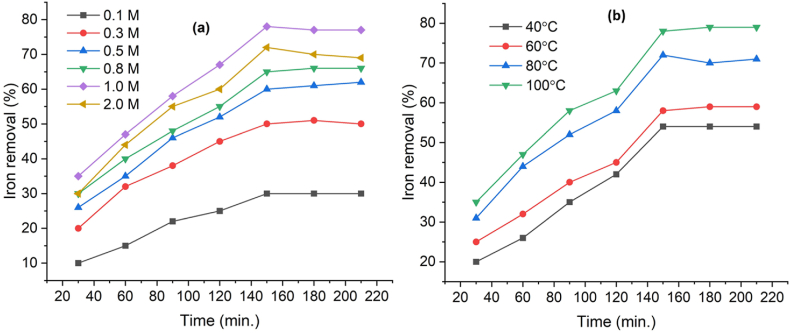
Effect of (a) oxalic acid concentrations and (b) temperatures on removal of iron during leaching.

The yield of clay increases linearly with the increasing concentration of oxalic acid, ranging from 0.1 to 2 M, over a period of 30–210 min (Fig. 4(a)). The removal of iron increased from 10 % to 78.71 % respectively. At an acid concentration of 2.0 M, approximately 72 % of the clay reacts after 150 min, potentially due to precipitation phenomena [29,30]. Thus, 1 M oxalic acid was determined to be the most effective concentration for iron removal, supporting the suitability of red clay for ceramic tile manufacturing. Fig. 4(b) shows the percentage of iron removal at different temperatures (40, 60, 80, and 100 °C) while maintaining a constant oxalic acid concentration of 1 M. As the temperature increased from 40 to 100 °C, the removal curves appeared parabolic, with slower oxidation rates at higher temperatures [31]. Consequently, the percentage of iron impurities removed increased with temperature [22]. From these observations, an oxalic acid concentration of 1.0 M, at 100 °C, and a leaching time of 150 min are optimal conditions for removing iron from red clay samples.

### 3.2 Effect of leaching on chemical composition

A comparative analysis was conducted based on XRF to evaluate the metal oxide content of raw red clay and red clay after leaching. After leaching, the composition of the red clay significantly changed, SiO_2_ increasing 53.6 %–63.13 %, Fe_2_O_3_ reducing 17.1 %–3.64 %, Al_2_O_3_ remaining relatively stable 18 %–18.22 % and other oxides more or less changed respectively (Table 2). However, Fe_2_O_3_ shows the maximum reduction at 13.46 % by weight, while SiO_2_ content increases by 9.53 % and 0.22 % increase in Al_2_O_3_ content because oxalic acid is the sole leaching agent, specifically targets Fe compounds without significantly interacting with SiO₂ or Al₂O₃. In addition, SiO₂ is chemically stable in the oxalic acid leaching process. That's why SiO₂ content has increased in absolute terms but why its proportion relative to the total mass of the clay has risen because the Fe₂O₃ content has been reduced. On the other hand the slight increase in Al₂O₃ content (from 0.22 %) could also be a result of similar concentration effects as observed with SiO₂ [[32], [33], [34]].

**Table 2:** Chemical composition between raw red clay and leached red clay.

Composition	Content (wt.%)	
	Before leaching	After leaching
SiO_2_	53.6	63.13
Al_2_O_3_	18	18.22
Fe_2_O_3_	17.1	3.64
K_2_O	5.07	6.2
Na_2_O	0.55	0.5
CaO	0.81	3.54
MgO	1.85	0.92
TiO_2_	1.99	2.99
P_2_O_5_	0.19	0.04
SO_3_	0.15	0.12
Cr_2_O_3_	0.03	0.03
MnO	0.14	0.24
CuO	0.02	0.01
NiO	0.03	0.02
ZnO	0.03	0.08
As_2_O_3_	0.009	0.005
Rb_2_O	0.06	0.03
SrO	0.03	0.02
ZrO_2_	0.17	0.07
Nb_2_O	0.0103	0.0141
BaO	0.121	0.143
Cl	0.053	0.08

However, Fe₂O₃ content was reduced, while SiO₂ content increased, which led to significant improvements in the aesthetic appeal and durability of the tiles, as well as enhance the efficiency of industrial tile manufacturing processes [35,36]. The stability of Al_2_O_3_ suggests that the leaching process primarily targets iron oxides without significantly affecting the aluminum content. Al_2_O_3_ is very important, mainly imparting stability to the melt and durability to the fired ceramics [29]. Notably, in the present research, Fe2O3 content (78.71 %) indicates the effectiveness of oxalic acid in removing iron impurities from the red clay, which is suitable for ceramic industries and supports our findings [22,36]. These changes in the chemical composition of the red clay post-leaching confirm the suitability of the treated clay for high-quality ceramic production, as the reduced iron content minimizes the risk of discoloration and improves the physical properties of the final ceramic products.

### 3.3 Effect of leaching parameters on iron concentration in leached solution

**Fig. 5:**
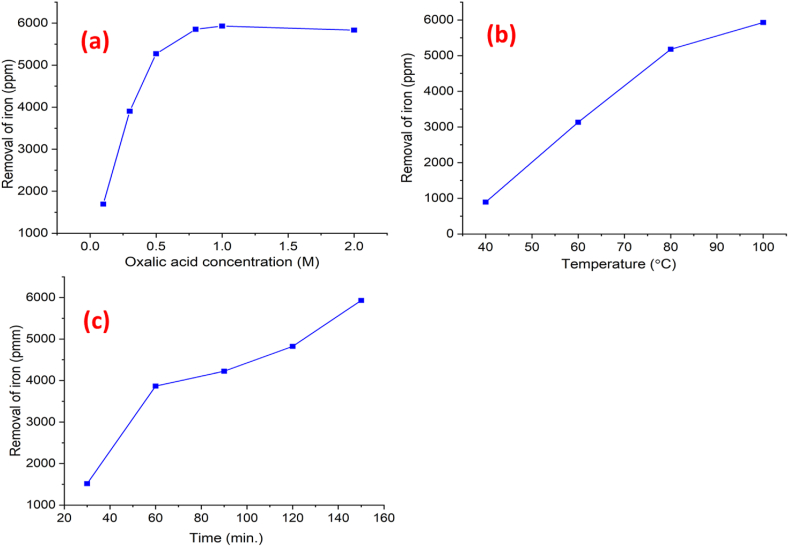
Iron removal efficiency based on (a) oxalic acid concentration, (b) temperature and (c) time.

Fig. 5 illustrates the effects of oxalic acid concentration, temperature, and reaction time on iron removal. In Fig. 5(a), the removal of iron increases linearly as the oxalic acid concentration rises from 0 to approximately 1.0 M, beyond which the removal rate plateaus, indicating a saturation effect at higher concentrations (up to 2.0 M). This suggests that after reaching an optimal concentration, further increases in oxalic acid do not significantly enhance iron removal efficiency. Similarly, Fig. 5(b) shows a consistent increase in iron removal as the temperature rises from 40 °C to 100 °C, with a nearly linear relationship, suggesting that higher temperatures positively impact the removal process by increasing reaction kinetics [37]. Lastly, Fig. 5(c) highlights the effect of reaction time, where iron removal progresses rapidly during the first 60 min, followed by a slower but steady increase up to 150 min, after which it reaches an equilibrium level, indicating a saturation effect as time progresses. The findings of this investigation confirm the efficiency of the oxalic acid leaching process for removing iron from red clay samples; it indicates 150 min, a temperature of 100 °C, and a 1.0 M concentration of oxalic acid are suitable for industrial applications, offering an effective method for iron removal from red clay.

### 3.4 Identify the red clay's crystalline phases

**Fig. 6:**
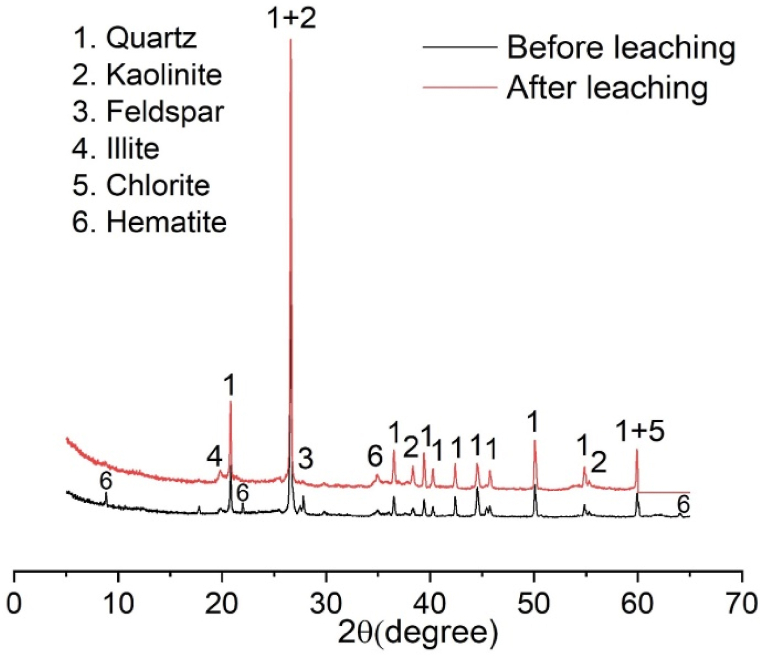
X-ray diffraction patterns of red clay before and after leaching.

The X-ray diffraction (XRD) analysis of red clay was conducted to identify its crystalline phases. The XRD patterns revealed that the primary mineral constituents of the raw red clay are illite, kaolinite, quartz, feldspar, hematite and chlorite (Fig. 6). These minerals contribute to the clay's overall properties, making it suitable for various industrial applications, including ceramics. Illite and kaolinite are clay minerals that provide plasticity and workability, which are essential for molding and shaping in ceramic manufacturing [38]. Quartz and feldspar, non-clay minerals, enhance the mechanical strength and thermal stability of the final ceramic products [39]. Feldspar increased the flexural strength and decreased the water absorption and linear shrinkage [40]. The presence of these minerals in the red clay samples indicates their potential utility in producing high-quality ceramics.

Post-leaching XRD analysis showed changes in the intensity of the diffraction peaks, reflecting the reduction in iron content and the overall improvement in clay purity. The reduction of iron impurities through oxalic acid leaching is evident from the diminished peaks corresponding to iron-bearing minerals. This enhanced purity of the leached red clay confirms its suitability for producing superior ceramic tiles. The removal of iron content from ceramic tiles has a significant impact on their color, strength, and thermal properties. However, reducing iron levels can enhance the quality and visual appeal of the tiles, as well as improve their longevity and thermal resistance [41,42].

### 3.5 Thermal analysis

**Fig. 7:**
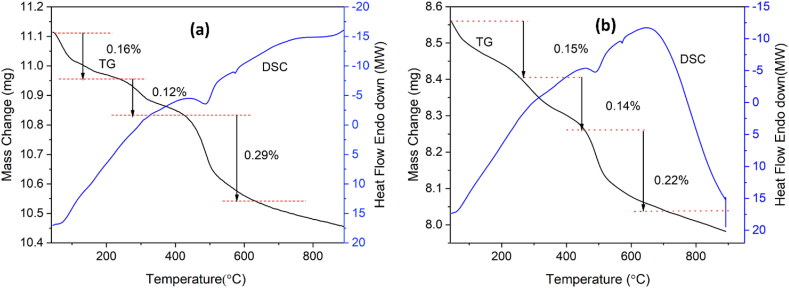
TG-DSC curves: **(a)** before leaching and **(b)** after leaching.

Thermogravimetric analysis (TG) and differential scanning calorimetry (DSC) were performed on red clay samples both before and after leaching to examine their thermal stability and weight loss characteristics. The TG-DSC curves for the raw red clay indicated three distinct weight loss regions. The initial weight loss, observed below 200 °C, was attributed to the removal of physically adsorbed water. The second weight loss occurred between 250 °C and 500 °C, corresponding to the loss of water molecules bound within the clay structure. The third weight loss, occurring between 500 °C and 750 °C, was due to the dehydroxylation of the clay minerals. Fig. 7 (a) (Before leaching) displays three endothermic peaks at 66.75, 486.66, and 574.21 °C from thermal analysis (TG-DSC) of fired red clay heated at 10 °C/min from 40 to 900 °C. Due to the evaporation of absorbed water, there was an initial weight loss of roughly 0.16 % below 200 °C, and chemically bonded water evaporated, resulting in a weight loss of 0.12 % from 236.6 °C to 424.67 °C. Finally, 0.29 % of weight loss was achieved from 424.67 °C to 632.57 °C, which represents the third endothermic peak. The literature review revealed that this region's clay exhibits comparable qualities (1, 2). After leaching, weight loss is almost the same as shown in Fig. 7 (b). After leaching with oxalic acid, the TG-DSC curves showed similar thermal behavior but with reduced weight loss percentages in the higher temperature ranges.

This reduction is primarily due to the removal of iron impurities, which can contribute to weight loss through thermal decomposition. The leached clay exhibited improved thermal stability, making it more suitable for high-temperature applications in the ceramic industry. The comparison of TG-DSC results before and after leaching demonstrates the effectiveness of the oxalic acid treatment in enhancing the thermal properties of the red clay. This improvement is critical for the production of high-quality ceramic products that require stability under high firing temperatures [29,30].

### 3.6 Surface morphology and elemental mapping

**Fig. 8:**
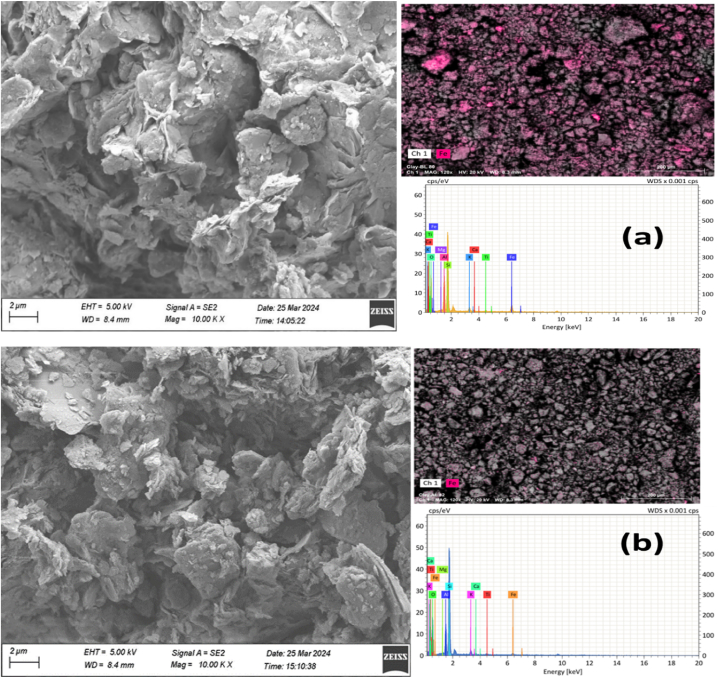
FESEM-EDX analysis of red clay (a) before leaching, (b) after leaching.

Field emission scanning electron microscopy (FESEM) images were captured to visualize the microstructural changes in red clay before and after iron removal through leaching (Fig. 8).

In the FESEM image taken before leaching Fig. 8(a), the presence of iron impurities is evident, appearing as bright sections scattered throughout the clay matrix. These impurities contribute to the rough and irregular surface texture of the raw red clay. In contrast, the FESEM image taken after leaching Fig. 8(b) shows a significant reduction in the bright sections, indicating the successful removal of iron impurities. The surface of the leached clay appears smoother and more uniform, reflecting the improved purity and quality of the clay post-treatment. This microstructural refinement is crucial for enhancing the performance of the clay in ceramic applications, ensuring better mechanical strength and aesthetic qualities in the final products [19].

### 3.7 Particle size distribution analysis

**Fig. 9:**
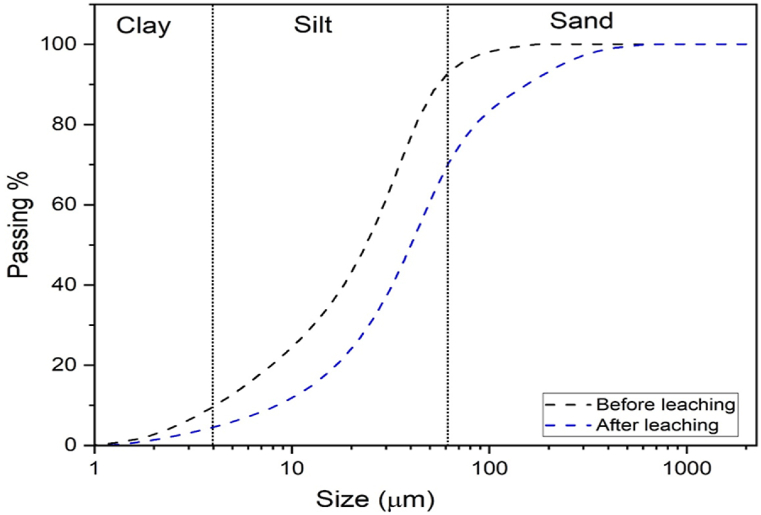
Particle size distribution analysis of red clay before and after leaching.

The comparison of particle size before and after the leaching of red clay using oxalic acid reveals significant changes that enhance its application in ceramic industries. Initially, red clay exhibits a varied particle size distribution with both fine and coarse particles, often agglomerated due to the presence of iron oxides. Red clay consists of silt (∼81 %), clay (<10 %), and sand (<9 %) which is suitable for ceramic manufacture for making ceramic tiles [43] (Fig. 9).

The leaching process, involving the application of oxalic acid, effectively dissolves these iron oxides, leading to the disintegration of larger agglomerates and resulting in a finer and more uniform particle size distribution. Red clay's grain size has an impact on its mechanical, technical, and microstructural characteristics throughout the drying and firing processes all of which are critical elements in the production of ceramics [44]. This reduction in particle size is crucial as it improves the clay's plasticity, surface finish, and mechanical strength, making it more suitable for high-quality ceramic products. Additionally, the removal of iron impurities through leaching minimizes defects such as discoloration and structural weaknesses, thereby enhancing the overall performance of the ceramic material. The transformation observed in the particle size distribution post-leaching underscores the efficacy of oxalic acid in refining red clay, thereby reinforcing its potential to produce superior ceramic products [45].

### 3.8 Suitability evaluation of leached red clay for preparing ceramics tiles

**Fig. 10:**
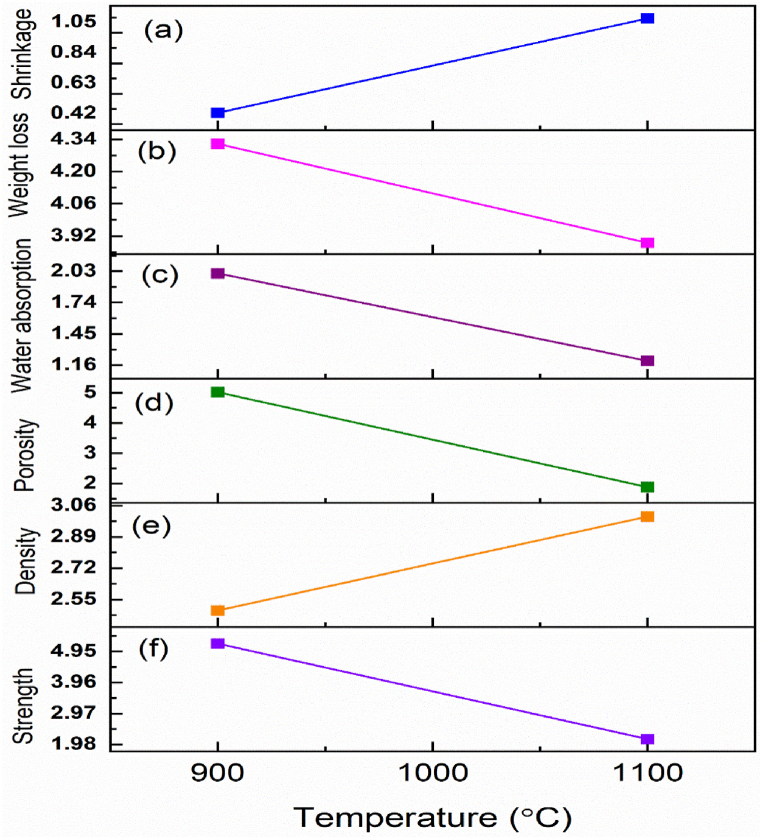
Analysis of the firing properties of ceramic qualities (a) linear shrinkage (%), (b) weight loss (%), (c) water absorption (%), (d) porosity (%), (e) bulk density (g/cm^3^), and (f) compressive strength (MPa).

The evaluation of ceramic qualities for mixed red clay tiles fired between 800 °C and 1100 °C reveals significant changes in their physical and mechanical properties. These properties include weight loss, porosity, bulk density, water absorption, and firing shrinkage, all of which are critical for determining the suitability of the tiles for various applications (Fig. 10). Firing shrinkage is another critical parameter affected by firing temperature. According to Fig. 10 and Table 3, the linear shrinkage of the tiles increases from 0.5 % at 900 °C to 1.15 % at 1100 °C. This increase is attributed to the rearrangement processes and the formation of a glassy phase during firing. Proper management of firing shrinkage is essential to avoid defects and ensure dimensional stability in the final product [46].

**Table 3:** Ceramic qualities of mixed red clay tiles burned between 900 °C and 1100 °C.

Firing properties	Temperature	
	900 °C	1100 °C
**Color**	White gray	White gray
**Sound**	Metallic	Metallic
**Linear shrinkage (%)**	0.5	1.15
**Weight loss (%)**	4.32	3.89
**Water Absorption, (%)**	2.01	1.2
**Apparent Porosity (%)**	5.01	1.88
**Bulk Density (g/cm**^**3**^**)**	2.49	3
**Unconfined compressive strength, (MPa)**	5.20	2.15

Weight loss in the tiles decreases with higher firing temperatures, primarily due to the removal of organic matter and other volatiles. This phenomenon is observed as weight loss decreases from 4.32 % at 900 °C to 3.89 % at 1100 °C. The removal of these components is essential for achieving the desired densification and mechanical strength in the final product [11].

The water absorption values exhibit a downward trend with increasing firing temperatures, decreasing from 2.01 % to 1.2 %. This reduction is closely linked to the densification process, which enhances the durability and resilience of the finished tiles [47]. Lower water absorption rates are crucial for producing high-quality ceramic products that meet industry standards [48]. Based on BS ISO 13006 (ISO, 2018) standards, ceramic tiles are classified into three groups according to their water absorption rates: Group I (<3 %), Group II (3%–10 %), and Group III (>10 %). The mixed red clay tiles examined in this study fall into Groups II and III, indicating their potential for use in vitrified or semi-vitrified tile applications [49]. With increasing firing temperature, the apparent porosity of the tiles decreases significantly, dropping from 5.01 % to 1.88 %. This reduction in porosity is due to the elimination of pore spaces through the evaporation of water and the burning of organic material. Consequently, this densification process also results in minor changes to the bulk density of the tiles, which typically ranges from 2.49 to 3 %. The apparent porosity of the tiles decreases significantly, dropping from 5.01 % to 1.88 %, with firing temperature increases. This reduction in porosity is due to the elimination of pore spaces through the evaporation of water and the burning of organic material. Consequently, this densification process also results in minor changes to the bulk density of the tiles, which typically ranges from 2.49 to 3 % [48,50].

The compressive strength of the tiles tends to decrease with higher firing temperatures, ranging from 5.20 MPa to 2.15 MPa [51]. This decrease may result from crystallization-recrystallization events occurring during the firing process. Despite this reduction, the absolute density remains below 3 g/cm³, making the material suitable for various industrial applications [52]. Post-leaching, the clay shows improved particle size distribution, resulting in finer particles that contribute to better plasticity, smoother surface finish, and higher mechanical strength in the final ceramic product. Additionally, the reduction in iron content minimizes the risk of defects, ensuring a more uniform and aesthetically pleasing tile. The refined red clay thus demonstrates excellent potential for ceramic tile applications, offering a viable and efficient raw material for producing tiles with superior quality and performance.

## 4 Conclusions

The study successfully demonstrates the efficacy of oxalic acid leaching in significantly reducing iron impurities from red clay, enhancing its suitability for use in the ceramic industry. Through the application of optimal leaching conditions (1.0 M oxalic acid concentration, 100 °C reaction temperature, 150 min of leaching time, and 250 rpm stirring), the iron content was reduced from 17.1 % to 3.64 %, achieving a removal efficiency of 78.71 %. This reduction aligns the treated clay's iron content with industry standards, making it a viable raw material for high-quality ceramic production. The comprehensive characterization of the red clay, involving particle size analysis, WDXRF, AAS, XRD, TGA, and FESEM, confirmed the improvement in chemical and physical properties post-leaching. Notably, the leached clay exhibited increased silica content (from 53.6 % to 63.13 %) and maintained a consistent alumina content, crucial for ceramic manufacturing. The mineralogical analysis identified the presence of beneficial phases such as illite, kaolinite, quartz, hematite, chlorite and feldspar, which contribute to the clay's enhanced mechanical properties and thermal stability. Furthermore, ceramic trials conducted at firing temperatures of 900 °C and 1100 °C demonstrated the treated clay's suitability for producing durable ceramic tiles. The tiles exhibited desirable properties such as low water absorption, high bulk density, and adequate compressive strength, aligning with industry standards for quality ceramic products.

Finally, this leaching process offers a practical solution for local ceramic industries in Bangladesh to use red clay as an alternative raw material. By effectively removing iron impurities, the process enhances the quality of red clay, making it suitable for ceramic production. Adopting this method could reduce the need for imported raw materials, thereby supporting local resource utilization and contributing to cost savings and sustainability in the industry.

## Future research direction

Future research should focus on implementing a pilot plant study to assess the industrial feasibility of the leaching process. Additionally, the iron oxalate present in the leached solution should be purified and further investigated for its potential applications in both scientific research and industry.

## Funding

This work was financially supported by 10.13039/501100005999Bangladesh Council of Scientific and Industrial Research (BCSIR), Bangladesh.

## CRediT authorship contribution statement

**Hayatullah:** Writing – original draft, Methodology, Investigation, Formal analysis, Conceptualization. **Asma Shafiq Shathi:** Methodology, Investigation, Conceptualization. **Md Golam Mostafa:** Writing – original draft, Software, Methodology, Investigation, Formal analysis. **Md Aminur Rahman:** Writing – review & editing, Supervision, Methodology, Formal analysis. **Pradip Kumar Biswas:** Writing – review & editing, Methodology. **Md Sha Alam:** Writing – review & editing, Formal analysis. **Md Shohel Rana:** Methodology, Formal analysis. **Md Ripaj Uddin:** Writing – review & editing, Writing – original draft, Validation, Methodology. **Md Nuruzzaman:** Writing – review & editing, Investigation, Formal analysis. **Md Shams Shahriar:** Resources, Conceptualization. **Mohammad Nazim Zaman:** Supervision, Project administration.

## Declaration of competing interest

The authors declare that they have no known competing financial interests or personal relationships that could have appeared to influence the work reported in this paper.
